# Thermodynamic Analysis for Binding of 4-*O*-β-tri-*N*-acetylchitotriosyl Moranoline, a Transition State Analogue Inhibitor for Hen Egg White Lysozyme

**DOI:** 10.3389/fmolb.2021.654706

**Published:** 2021-06-10

**Authors:** Makoto Ogata, Tamo Fukamizo, Takayuki Ohnuma

**Affiliations:** ^1^ Faculty of Food and Agricultural Sciences, Fukushima University, Fukushima, Japan; ^2^ Department of Advanced Bioscience, Kindai University, Nara, Japan; ^3^ Agricultural Technology and Innovation Research Institute(ATIRI), Kindai University, Nara, Japan

**Keywords:** 4-O-β-tri-N-acetylchitotriosyl moranoline, lysozyme (HEWL), thermodynamics, inhibitor, binding

## Abstract

4-*O*-*β*-tri-*N*-acetylchitotriosyl moranoline (GN_3_M) is a transition-state analogue for hen egg white lysozyme (HEWL) and identified as the most potent inhibitor till date. Isothermal titration calorimetry experiments provided the thermodynamic parameters for binding of GN_3_M to HEWL and revealed that the binding is driven by a favorable enthalpy change (Δ*H*° = −11.0 kcal/mol) with an entropic penalty (−*T*Δ*S*° = 2.6 kcal/mol), resulting in a free energy change (Δ*G*°) of −8.4 kcal/mol [Ogata et al. (2013) 288, 6,072–6,082]. Dissection of the entropic term showed that a favorable solvation entropy change (−*T*Δ*S*_solv_° = −9.2 kcal/mol) is its sole contributor. The change in heat capacity (Δ*C*_p_°) for the binding of GN_3_M was determined to be −120.2 cal/K·mol. These results indicate that the bound water molecules play a crucial role in the tight interaction between GN_3_M and HEWL.

## Introduction

Lysozyme (EC 3.2.1.17) is an enzyme that hydrolyzes the β-1,4-glycosidic bond between *N*-acetylmuramic acid and *N*-acetylglucosamine (GlcNAc) in peptidoglycan, a major structural component of the bacterial cell wall (Jollès and Jollès, 1984). Hen egg white lysozyme (HEWL) is the first enzyme to have its three-dimensional structure determined by X-ray diffraction (Blake et al., 1965), consequently its catalytic mechanism has been intensively studied. Based on the modeled structure of HEWL-chitohexaose, it was proposed that HEWL has six subsites for binding of sugar residues in the active site cleft, termed −4 to +2 (formerly A, B, C, D, E, and F), and the cleavage occurs between sugars located at subsites −1 and +1 through the cooperative action of Glu35 and Asp52 (Johnson and Phillips, 1965; Blake et al., 1967; Phillips, 1967). In the catalytic reaction, Glu35 is thought to act as a general acid catalyst to protonate the glycosidic oxygen, while Asp52 acts as a conjugate base and stabilizes the carbonium ion intermediate that adopts a half-chair conformation with C1 carbon displaying *sp* ^2^ hybridization. This is known as the Phillips mechanism, widely supported by a number of experimental observations including mutagenesis studies of these amino acids (Malcolm et al., 1989).

**FIGURE 1:**
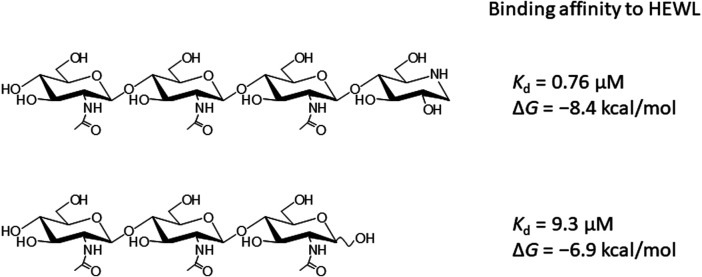
Chemical structure of GN_3_M and (GlcNAc)_3_.

Meanwhile, Vocadlo et al. reported the crystal structure of HEWL, covalently bound to C1 carbon of the −1 sugar, which exhibits a chair conformation with C1 carbon in *sp* ^3^ hybridization (Vocadlo et al., 2001). Recently, we synthesized 4-*O*-β-tri-*N*-acetylchitotriosyl moranoline (GN_3_M) from chitoteratetraose (GlcNAc)_4_ and moranoline with the aid of the lysozyme-catalyzed transglycosylation reaction and examined its inhibitory action toward HEWL (Figure 1) (Ogata et al., 2013). GN_3_M with C1 carbon in *sp* ^3^ hybridization was found to be the most potent lysozyme inhibitor till date with an inhibition constant *K*_i_ of 1.84 μM and bind tightly to HEWL (*K*_d_ = 0.76 μM at 25°C). Furthermore, in the HEWL-GN_3_M complex structure, GN_3_M was well superimposed on NAG2FGlcF ((GlcNAc)_2_-fluoro-glucosyl fluoride), covalently bound to HEWL mutant E35Q and the moranoline moiety bound to subsite −1 was in a chair conformation (without distortion) as the −1 sugar of the covalently bound NAG2FGlcF. From these results, we concluded to support the covalent glycosyl-enzyme intermediate formation in the reaction catalyzed by the wild-type HEWL. This is known as the Koshland mechanism, now more widely accepted by enzyme researchers (Koshland, 1953).

In this study, in order to elucidate the driving forces responsible for the tight binding of GN_3_M (a transition-state analogue inhibitor) to HEWL, we conducted a detailed thermodynamic analysis using isothermal titration calorimetry. The thermodynamic data obtained from our study would be useful to understand the substrate binding mechanism of HEWL and to design novel glycosidase inhibitors with moranoline moiety.

## Materials and Methods

### Materials

4-*O*-β-tri-N-acetylchitotriosyl moranoline (GN_3_M) was prepared by lysozyme-mediated transglycosylation from the substrates tetra-*N*-acetylchitotetraose (GlcNAc)_4_, and moranoline (1-deoxynojirimycin) as described previously (Ogata et al., 2013). HEWL, which was recrystallized six times, was purchased from Seikagaku Kogyo Co. (Tokyo, Japan). All other reagents were of the highest quality commercially available and were used without further purification.

### Isothermal Titration Calorimetry (ITC) Experiments

The HEWL solution (45 μM) in 20 mM phosphate buffer (pH 7.0) was degassed and its concentration was determined by measuring the absorbance of ultraviolet light at 280 nm. GN_3_M was dissolved (0.5 mM) in 20 mM phosphate buffer (pH 7.0), degassed, and loaded into a syringe, whereas the HEWL solution (0.2028 ml) was loaded into the sample cell. Calorimetric titration was performed with an iTC_200_ system (Microcal Northampton, MA, United States). For the titrations, 2.5 μl of a ligand was injected into the sample cell at an interval of 180 s with a stirring speed of 1,000 rpm. The heat of dilution caused by an injection of GN_3_M was measured under identical buffer, injection, and temperature conditions but by adding ligand to a sample solution that lacked protein. The heat of dilution was subtracted from the heat change that occurred in presence of the protein. Origin® software was used to analyze the ITC data. Using the single-site binding model, individual datasets obtained from the titration experiments fitted well to the theoretical curves, providing stoichiometries (*n*), equilibrium binding association constants (*K*_a_), and enthalpy changes (Δ*H*°) of the protein-ligand interactions. The value of *n* was found to be between 1.02 and 1.22 for all titrations. The binding free energy change (Δ*G*°) and the entropy change (Δ*S*°) were calculated from the relationship described in Eq. 1.

$$\Delta G{}^{\circ}=-\mathrm{RT}\cdot \text{ln}\;K_{a}=\Delta H{}^{\circ}-Τ\Delta S{}^{\circ}$$ (1)

To examine temperature dependence, ITC measurements were performed at pH 7.0 and the temperature was varied in five-degree increments from 15 to 35°C. Methods for obtaining the heat capacity change (Δ*C*_p_∘) and parameterizing the entropic term have been described previously (Ohnuma et al., 2011). Briefly, the binding heat capacity change (Δ*C*_p_°) was obtained from the ITC titrations over temperature range. Measurements of temperature dependence of Δ*H*° for GN_3_M binding to HEWL in the temperature range yielded a straight line of slope equal to Δ*C*_p_°. Errors are reported as standard deviations of at least three experiments at each temperature.

## Results and Discussion

**FIGURE 2:**
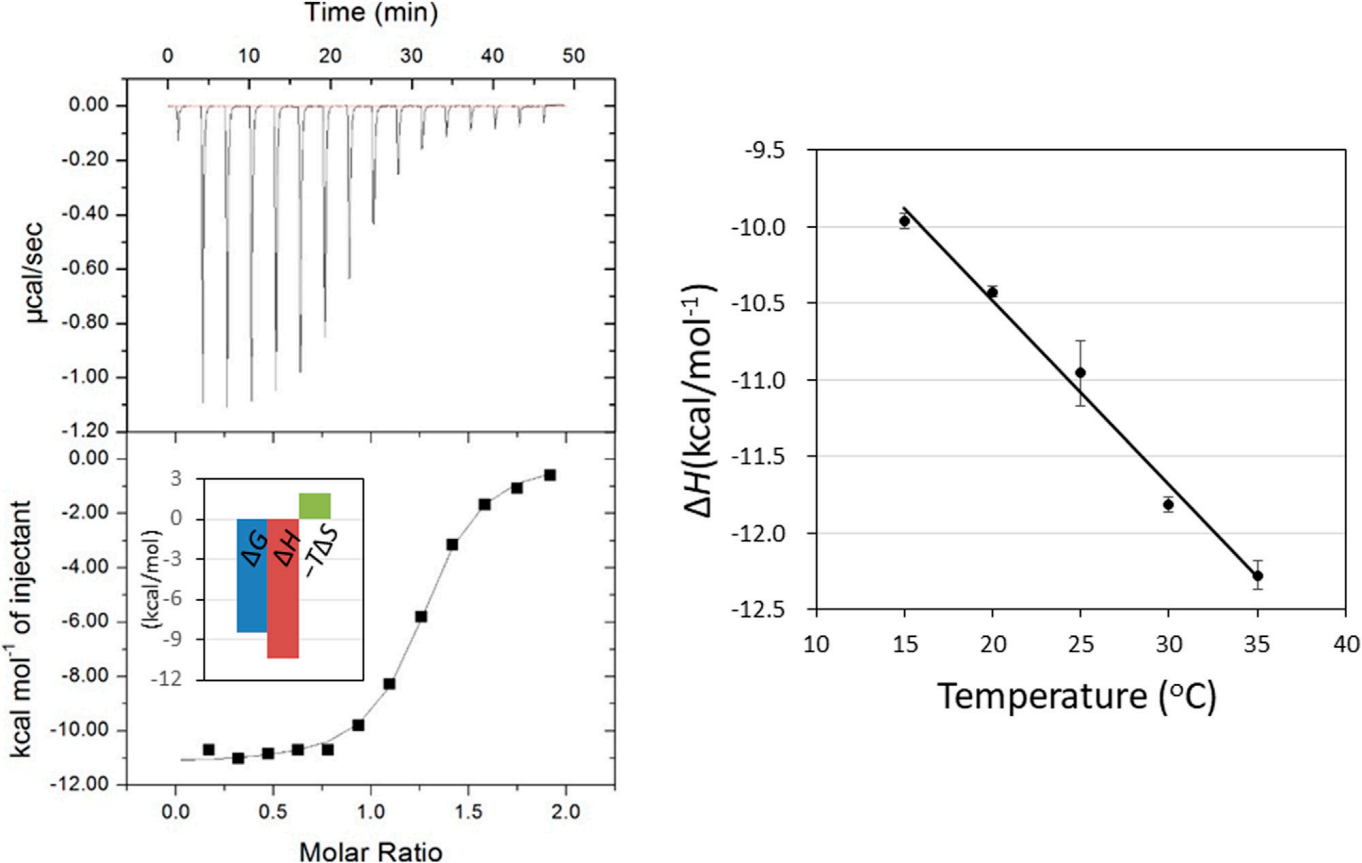
ITC thermogram **(upper)** and theoretical fit to the experimental data **(lower)** for binding of GN_3_M at 20°C. Inset shows the bar diagram of the thermodynamic parameters **(A)**. Temperature dependence of GN_3_M binding to HEWL; the plot of Δ*H*° versus temperature yielded a change in the heat capacity (Δ*C*_p_°) based on the slope of the line. The Δ*C*_p_° value was calculated to be −120.2 cal/*K*·mol **(B)**.

**FIGURE 3:**
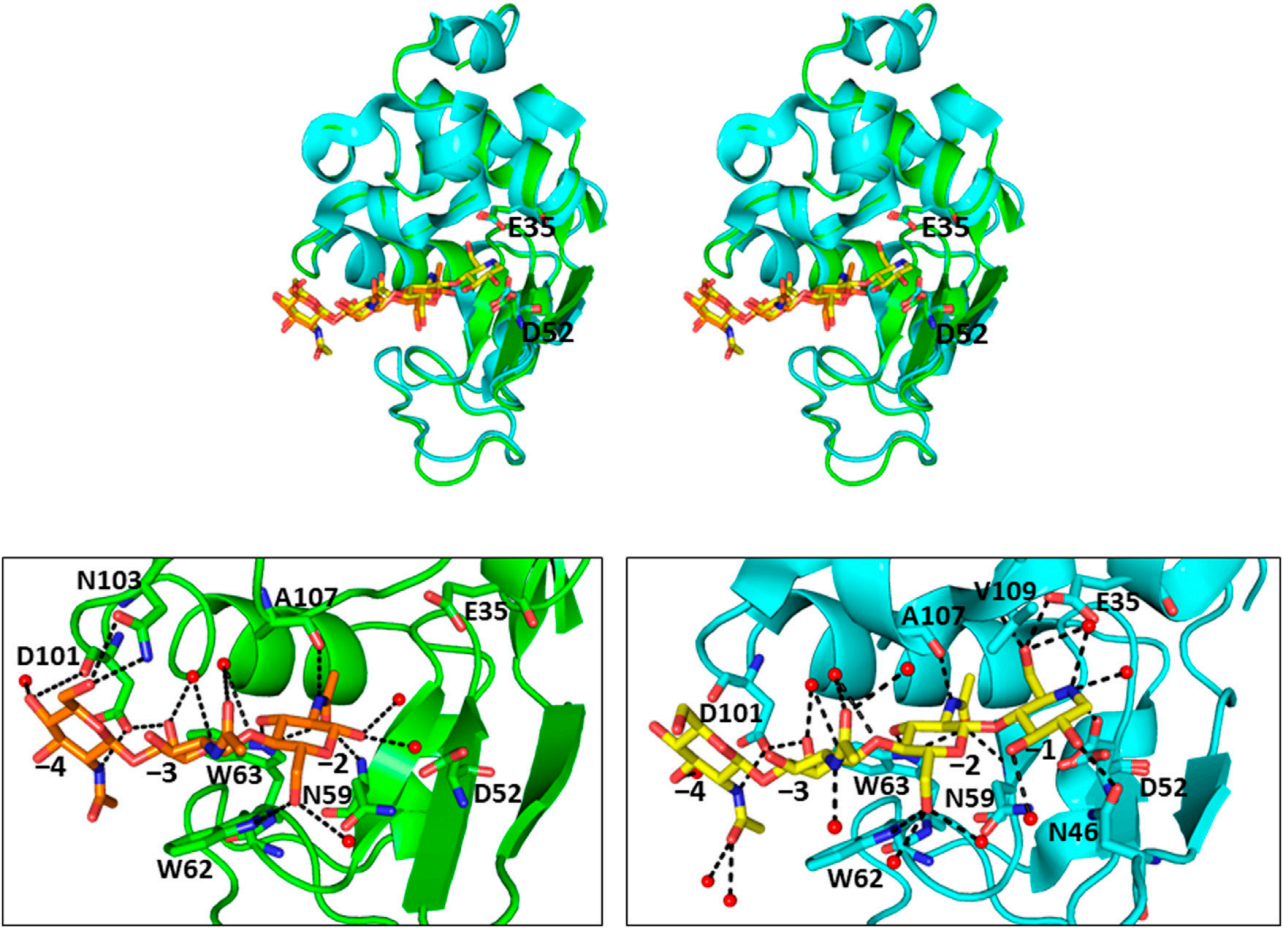
Crystal structures of (GlcNAc)_3_-liganded and GN_3_M-liganded HEWL **(A)** Stereo view of superimposed structures of (GlcNAc)_3_-liganded HEWL (green; PDB code 1lzb) and GN_3_M-liganded HEWL (cyan; PDB code 4hp0) complexes. The catalytic residues Glu35 and Asp52 of HEWL are indicated as sticks (GlcNAc)_3_ and GN_3_M are shown as orange and yellow sticks, respectively. **(B)** The binding modes of (GlcNAc)_3_ and GN_3_M to HEWL. Amino acid residues involved in the binding of ligands (GlcNAc)_3_ and GN_3_M are also indicated as sticks. The numbers, −4 to −1, indicate the subsite positions. Dashed lines indicate the possible hydrogen bonds. Red spheres represent oxygen atoms of water molecules.

In addition to chitin oligosaccharides (GlcNAc)_n_ (*n* = 2–3), we previously investigated the inhibitory effects of 4-*O*-*β*-tri-*N*-acetylchitotriosyl-2-acetamido-2,3-dideoxydidehydroglucopyranose (GN_3_D), 4-*O*-*β*-tri-*N*-acetylchitotoriosyl-2-acetamido-2,3-dideoxydidehydroglucono-δ-lactone (GN_3_L), and 4-*O*-*β*-tri-N-acetylchitotriosyl moranoline (GN_3_M) against HEWL (Figure 1). GN_3_M was found to be the most potent inhibitor (*K*_i_ = 1.84 μM). Based on the thermodynamic parameters provided by ITC experiments for HEWL binding of these compounds, GN_3_M was found to be a tight binding inhibitor (Δ*G*° = −8.4 kcal/mol; *K*_d_ = 0.76 M) (Ogata et al., 2013). Figure 2A shows a typical ITC thermogram and theoretical fit to the experimental data for binding of GN_3_M at 20°C. Figure 3A shows superimposed structures of (GlcNAc)_3_-liganded HEWL (PDB code 1lzb) and GN_3_M-liganded HEWL (PDB code 4hp0) complexes (Maenaka et al., 1995; Ogata et al., 2013). The main chain of GN_3_M-liganded HEWL overlapped well with that of (GlcNAc)_3_-liganded HEWL with a RMS deviation of 0.235 Å in the superimposition of the corresponding 128 Cα-atoms. The (GlcNAc)_3_ moiety of GN_3_M bound to HEWL also overlapped with (GlcNAc)_3_ bound to HEWL. All saccharide rings in the complex structures were in the ^4^ *C*_1_ chair conformation with small variations. Thus, the potent inhibitory activity and tight binding ability of GN_3_M (a (GlcNAc)_3_-moranoline conjugate) towards HEWL, in comparison to other compounds may be attributed to the moranoline moiety of GN_3_M. The Δ*G*° value for (GlcNAc)_3_ binding to HEWL was found to be −6.9 kcal/mol. In this case, (GlcNAc)_3_ binds to the subsites −4, −3, and −2 of HEWL, as shown in the crystal structure of the HEWL-(GlcNAc)_3_ complex (Figure 3B, left). Therefore, it appears that moranoline residue attached to the reducing end of (GlcNAc)_3_ contributes to the binding free energy of −2.5 kcal/mol (ΔΔ*G*° = −8.4 − (−6.9) kcal/mol), resulting in a 12.2-fold enhancement in the binding affinity (*K*_d_ of 0.76 μM for GN_3_M and 9.3 μM for (GlcNAc)_3_). Williams et al. synthesized the xylobio-deoxynojirimycin analogue (a xylanase inhibitor and xylose-moranoline conjugate) and demonstrated that it binds to the retaining family of 10 xylanase Cex from *Cellulomonas fimi* approximately 830-fold more tightly than xylobiose (*K*_i_ of 5.8 μM for xylobio-deoxynojirimycin analogue and 4,800 μM for xylobiose) (Williams et al., 2000). Arai et al. synthesized (glucose)_n_-deoxynojirimycin (*n* = 1–8) conjugates by the transglucosylation action of bacterial saccharifying amylases and showed that these compounds have inhibitory activity against α- and β-amylases of various origins (Arai et al., 1986). Therefore, conjugation of moranoline to the reducing end site of mono- or oligosaccharides is an excellent strategy to design potent inhibitors of the target glycosidases. Since GN_3_M lacks an acetamido group at the C2 of the moranoline moiety bound to the −1 subsite of HEWL (Figure 3B, right), conjugation of *N*-acetylmoranoline to the reducing end of (GlcNAc)_3_ could improve the inhibitory activity of (GlcNAc)_3_ (de la Fuentea et al., 2016).

Determination of temperature dependence of Δ*H*° for binding of GN_3_M at pH 7.0 in the temperature range of 15–35°C, yields a change in the reaction heat capacity (Δ*C*_p_°) (Figure 2B). The Δ*C*_p_° value for GN_3_M binding to HEWL was found to be −120.2 cal/*K*·mol (Table 1). Negative heat capacity changes are often attributed to the release of well-ordered water molecules from the interface between the protein and the ligand into a bulk solvent by forming hydrophobic contacts. Therefore, we decomposed the entropic terms for binding of GN_3_M to HEWL as follows. By recognizing that the entropy of solvation is close to zero for proteins near 385 K, Δ*C*_p_° can be related to the solvation entropy change (Δ*S*_solv_°) of the binding reaction at t = 25°C as described in Eq. 2 (Baldwin, 1986; Murphy et al., 1990; Baker and Murphy, 1997).

ΔSsolv°=ΔCp° ln(T298 K/T385 K) (2)

Furthermore, the mixing entropy change (Δ*S*_mix_°) is a statistical correction that reflects mixing of solute and solvent molecules and the changes in translational/rotational degrees of freedom as described in Eq. 3 (Baker and Murphy, 1997).

$$\Delta S_{\mathrm{mix}}{}^{\circ}=R\ln\left(1/55.5\right)=-8\;\mathrm{cal}/K\cdot \mathrm{mol}$$ (3)

**TABLE 1:** Parameterization of the entropic term for binding of GN_3_M to HEWL at 25°C.

Inhibitor Δ*C*_p_°^a^ (cal∙K^−1^∙mol^−1^)	*−T*Δ*S*° (kcal∙mol^−1^)	*−T*Δ*S*_mix_°^b^ (kcal∙mol^−1^)	*−T*Δ*S*_solv_°^c^ (kcal∙mol^−1^)	*−T*Δ*S*_conf_°^d^ (kcal∙mol^−1^)
GN_3_M –120.2	2.6	2.4	–9.2	9.3

a Data are derived from the temperature dependence of Δ*H*°.

b Δ*S*_mix_° = *R*ln (1/55.5) = −8 cal/K mol (Baker and Murphy, 1997).

c Δ*S*_solv_° = Δ*C*_p_ ln (*T*_298 K_/*T*_385 K_) (Baldwin, 1986; Murphy et al., 1990; Baker and Murphy, 1997).

d Derived using Δ*S*° = Δ*S*_solv_° + Δ*S*_mix_° + Δ*S*_conf_° (Baker and Murphy, 1997).

The conformational entropy change (Δ*S*_conf_°) details the change in side-chain and backbone conformational entropy associated with binding. The reaction entropy change (Δ*S*°), which is derived from the ITC experiment, can be viewed as the sum of Δ*S*_solv_°, Δ*S*_mix_° and Δ*S*_conf_° (Baker and Murphy, 1997). The results summarized in Table 1 show that at pH 7.0 − *T*Δ*S*_solv_° is equal to −9.2 kcal/mol (Δ*S*_solv_° = 30.8 cal/*K*·mol), −*T*Δ*S*_mix_° = 2.4 kcal/mol (Δ*S*_mix_° = −8.0 cal/*K*·mol) and −*T*Δ*S*_conf_° is equal to 9.3 kcal/mol (Δ*S*_conf_° = −31.1 cal/*K*·mol). As shown in Table 1, while the −*T*Δ*S*_solv_° value for binding was negative (−9.2 kcal/mol), the −*T*Δ*S*_conf_° value was positive (9.3 kcal/mol), resulting in a net entropic penalty of 2.6 kcal/mol. These results indicate that the favorable solvation entropy change is the only contributor in the entropic term. A negative value of −*T*Δ*S*_solv_° (positive value of Δ*S*_solv_°) implies water molecules are expulsed upon ligand binding due to hydrophobic interactions. Although the precise information regarding the solvation state of HEWL before and after binding to GN_3_M is not available, upon GN_3_M binding to HEWL, the apolar solvent accessible surface area (*ASA*_apolar_) was reduced with over 7%. On the other hand, the same difference in *ASA*_apolar_ between (GlcNAc)_3_-bond and ligand free HEWL was negligibly small (calculated using GetArea 1.1, TX, United States, based on the crystal structures to ligand-bound and ligand-free HEWL) (Table 2) (Fraczkiewicz and Braun, 1998). Figure 3B shows the binding modes of (GlcNAc)_3_ and GN_3_M to HEWL. Compared to the HEWL-(GlcNAc)_3_ complex structure, more water molecules were observed at HEWL-GN_3_M interfaces. However, the solvation entropy change upon GN_3_M binding to HEWL is favorable (positive) (Table 1). Therefore, it is likely that dehydration for GN_3_M binding involves the ligand, the active site, or, in case of conformational changes upon binding, the bulk of protein.

**TABLE 2:** The solvent accessible surface areas (*ASAs*) of ligand-bound and ligand-free HEWL.

Structures	*ASA*_apolar_ (Å^2^)	*ASA*_polar_ (Å^2^)	*ASA*_total_ (Å^2^)
HEWL	3,628	2,850	6,478
HEWL-GN_3_M	3,172	2,598	5,771
HEWL-(GlcNAc)_3_	3,690	2,875	6,566

GetArea 1.1 software was used for calculating the *ASAs* from the crystal structures of HEWL (PDB ID: 1lzd), GN_3_M-liganded HEWL (PDB ID: 4hp0) and (GlcNAc)_3_-liganded HEWL (PDB ID: 1lzb). This program separates the solvent accessible surface area (*ASA*_total_) into the apolar (*ASA*_apolar_) and polar (*ASA*_polar_) components.

In this study, the thermodynamic analysis for binding of GN_3_M yielded valuable information regarding the driving forces behind the tight binding of this inhibitor. In addition to the enthalpic contribution, the favorable solvation entropy change is the only contributor in the entropic term. Since they mimic the transition states of the hydrolytic reactions, iminosugar-conjugated glycosidase inhibitors containing moranoline residues have been designed and synthesized (Arai et al., 1986; Notenboom et al., 2000; Williams et al., 2000; Kato et al., 2011). They are known to have potent inhibitory activity against the target glycosidases. Therefore, it would be relevant to consider their thermodynamic properties for binding to the target enzymes, especially the solvation entropy change, in the rational design of novel inhibitors with improved properties.

## Data Availability

The raw data supporting the conclusions of this article will be made available by the authors, without undue reservation.

## Abbreviations

GlcNAc, *N*-acetyl-β-d-glucosamine; (GlcNAc)_*n*_, *β*-1,4-linked oligosaccharide of GlcNAc with a polymerization degree of n; GN_3_M, 4-*O*-*β*-tri-*N*-acetylchitotriosyl moranoline; HEWL, hen egg white lysozyme; ITC, isothermal titration calorimetry; NAG2FGlcF, 2-Acetamido-2-deoxy-β-D-glucopyranosyl-(1→4)-2-deoxy-2-fluoro-β-D-glucopyranosylfluoride.
